# A Rare Cause of Primary Aortoenteric Fistula:* Streptococcus parasanguinis* Aortitis

**DOI:** 10.1155/2017/9087308

**Published:** 2017-01-31

**Authors:** Fredy Nehme, Kyle Rowe, Cyrus Munguti, Imad Nassif

**Affiliations:** Department of Internal Medicine, Kansas University School of Medicine, Wichita, KS, USA

## Abstract

Primary aortoenteric fistula is a rare cause of upper gastrointestinal bleed but can lead to significant mortality if the diagnosis is delayed. Aortitis, characterized by inflammation of the aortic wall, is a rare cause of aortoenteric fistula. We present a case report of a 72-year-old male patient with infectious aortoenteric fistula secondary to* Streptococcus parasanguinis*, along with a review of the literature. This case demonstrates the importance of early diagnosis and aggressive surgical treatment of aortoenteric fistulae and recognizing infectious aortitis as a potential etiology.

## 1. Introduction

Primary aortoenteric fistula (PAEF) is a direct communication between the aorta and the intestinal lumen without prior reconstructive procedures on the abdominal aorta. Although rare with an estimated prevalence of 0.07% [1], PAEF is a severe life threatening emergency that should be promptly recognized. Urgent surgical repair is usually warranted. PAEF commonly predominates in the third and fourth portion of the duodenum [2]. The leading causes of PAEF are atherosclerosis and aortic aneurysm in about 70% of cases, while aortitis remains a less common etiology [1]. In this report, we describe a rare case of PAEF caused by infective aortitis due to* Streptococcus parasanguinis*. Early recognition and urgent surgical repair lead to a favorable outcome.

## 2. Case Presentation

**Figure 1:**
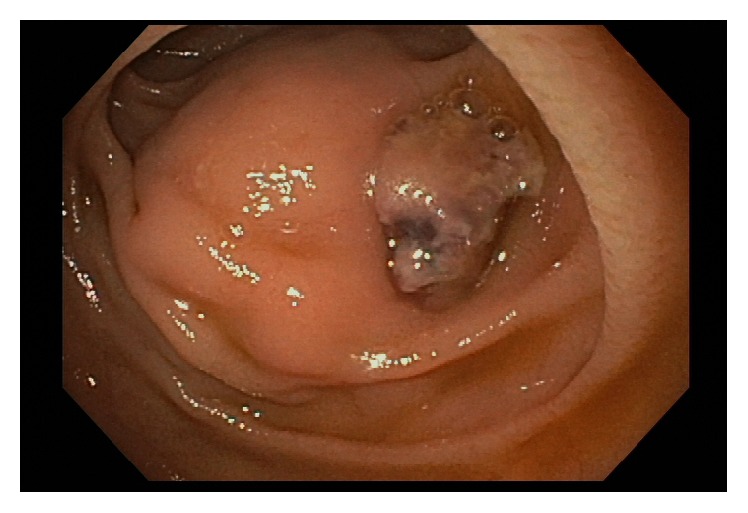
EGD showing a large pulsating bulge in the second portion of the duodenum with an adjacent 15 mm ulcer.

**Figure 2:**
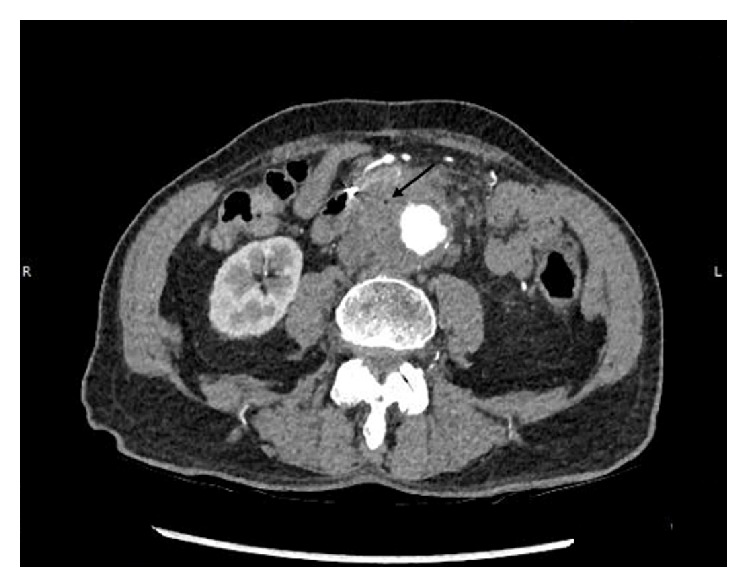
CTA showing a 5.3 cm aortic aneurysm, marked soft tissue thickening of the aortic wall, and a small pocket of gas within the soft tissue of the aorta (soft tissue gas designated by the arrow).

A 72-year-old male presented with chief complains of acute onset abdominal pain, back pain, and hematemesis. The patient reported vomiting around 2 ounces of blood followed by an episode of melena. He reported chills, fatigue, diaphoresis, and subjective fevers for the last week. He denied chest pain, shortness of breath, or cough. His past medical history was significant for a right lower lobe stage IB non-small cell lung cancer treated with radiation therapy currently in remission and polyangiitis obliterans with resultant upper and lower limb amputations. Physical examination revealed a temperature of 99.5 F, with a regular pulse rate of 74/min and a blood pressure of 136/83 mmHg. Abdominal exam elicited mild diffuse tenderness without guarding. Laboratory investigation was significant for anemia with hemoglobin of 8 g/dL. Inflammatory markers were elevated with a C-reactive protein of 12.6 mg/dL and erythrocyte sedimentation rate of 110 mm/h. No coagulopathy or thrombocytopenia was noted. Blood cultures were not drawn. Esophagogastroduodenoscopy (EGD) revealed a large pulsating bulge in the second portion of the duodenum. A 15 mm ulcer with an adherent clot was also visualized in the same region without active bleeding (Figure 1). Urgent computed tomography angiography (CTA) showed a 5.3 cm aortic aneurysm, marked soft tissue thickening of the aortic wall, and a small pocket of gas within the soft tissue of the aorta near the second portion of the duodenum (Figure 2). With findings suspicious of aortitis and aortoenteric fistula, vascular surgery was consulted and urgent exploratory laparotomy was scheduled. Upon exploration, adhesions between the small bowel, the omentum, and the anterior abdominal wall were carefully taken down. There was clear evidence of a fistula with adherence of the small bowel to the anterior lateral aneurysm wall. The aneurysmal portion involving the fistula was noted to contain significant purulence requiring surgical debridement. The involved aortic aneurysm was resected. A 16-mm Hemashield tube graft was sewn between the infrarenal aorta and distal aorta with 3-0 Prolene sutures. A duodenojejunostomy with resection of the involved fourth portion of the duodenum was then performed.

**Figure 3:**
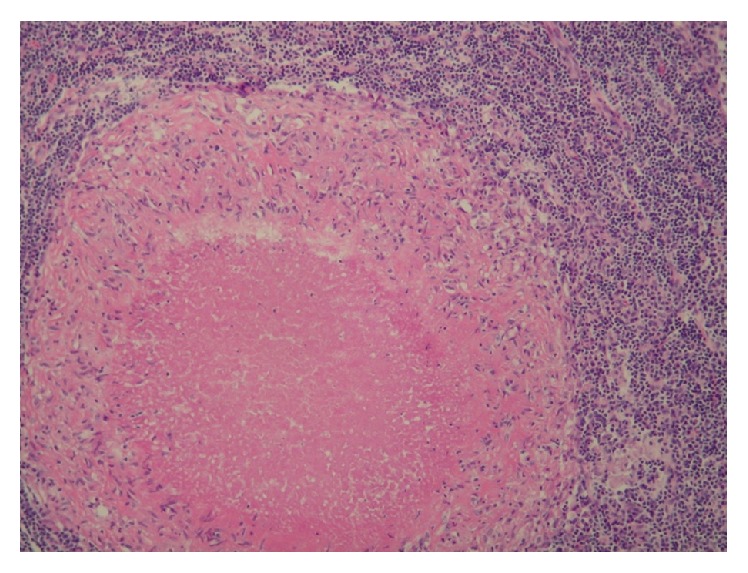
Surgical pathology revealing intense active inflammation with the presence of necrotizing granulomas.

A mycotic aneurysm was strongly suspected and the patient was started empirically on Meropenem. Cultures from the excised aortic aneurysm were positive for* Streptococcus parasanguinis* and antibiotics were deescalated appropriately. Acid-fast bacilli and fungal stains were negative. Surgical pathology revealed intense chronic active inflammation with the presence of necrotizing granulomas (Figure 3). Resection margins were free of active inflammation. A 6-week course of antibiotics was completed with significant improvement. Follow-up at 6 months demonstrated complete resolution of symptoms.

## 3. Discussion

Aortoenteric fistula (AEF), defined as an abnormal connection between the aorta and the gastrointestinal tract, is a rare cause of upper gastrointestinal bleeding but can lead to significant morbidity and mortality if not promptly recognized. Two types of AEFs are described, primary aortoenteric fistula arising de novo between the aorta and the bowel or secondary aortoenteric fistula (SAEF) occurring after aortic reconstruction surgery. SAEF is far more common [3].

The “herald bleed” is a common presentation of AEF that needs to be recognized early in order to prevent a later catastrophic hemorrhage. The diagnosis of PAEF is elusive as the classic triad of gastrointestinal bleeding, abdominal pain, and palpable mass is only found in 11% of patients [2] and upper gastrointestinal endoscopy has a sensitivity of only 50% [4]. It is important to recognize the endoscopic findings suggestive of AEF, particularly the presence of a pulsating mass, as dislodging a fresh thrombus may lead to massive hemorrhage. CTA is currently the first line imaging modality to evaluate an AEF bleed [4].

Aortitis, characterized by inflammation of the aortic wall [5], is an uncommon cause of aortic aneurysm with inflammatory aneurysms compromising only 3 to 10% of abdominal aortic aneurysms [6]. The most common causes of aortitis include the large vessel vasculitis, giant cell arteritis, and Takayasu arteritis. Other rheumatologic diseases are also associated with aortitis [5]. Infectious aortitis is a rare but life threatening condition that must be differentiated from other inflammatory conditions as treatment strategies diverge widely [5]. Bacterial seeding of the aortic wall, usually with an underlying pathology, occurs via the vasa vasorum [7]. This results in the formation of an aneurysm or pseudoaneurysm that erodes into the adjacent structures [8]. In our case, the diagnosis of aortitis was strongly supported by elevated inflammatory markers, CTA findings of marked soft tissue thickening of the aortic wall, and pathology results demonstrating intense active inflammation with the presence of necrotizing granulomas. The intraoperative finding of significant purulent material around the involved aortic segment, the presence of periaortic gas on CTA, and the isolation of a bacterial organism were in favor of an infectious etiology which prompted the initiation of broad spectrum antibiotics. However, the presence of periaortic gas can also be attributed to presence of an AEF. Although* Streptococcus parasanguinis,* a member of the viridans group, is associated with a variety of infections, mycotic aneurysm is uncommon. In our case, the source of infection was unknown.

Therapy of infected aneurysms relies mainly on a combination of antibiotic therapy, surgical debridement, and, if possible, revascularization [9]. Timing and type of surgical repair depend on multiple factors including severity of the clinical presentation, patient comorbidities and type of AEF. Open surgical repair generally involves vascular control of the aorta followed by debridement of all infected and necrotic tissue, repair or resection of the intestinal defect, and in situ aortic reconstruction with a prosthetic graft [9]. While revascularization is usually performed immediately in PAEF, the timing and type of revascularization are debated in SAEF. For patients who are not considered good surgical candidates for open repair, an endovascular approach is suggested either as a bridge procedure to definitive repair [10] or as an adjunct to palliative treatment [11]. Options include endovascular balloon occlusion of the aorta, endovascular coil embolization, and stent-graft repair [12]. Optimal antibiotic duration is uncertain and varies depending on several factors. Usually, six weeks of antibacterial therapy is suggested [13].

## 4. Conclusion

Early diagnosis and aggressive surgical treatment of an AEF are vital to achieve a positive outcome. Aortitis is a rare cause of AEF that should be considered based on clinical, imaging, and surgical findings. Infectious aortitis, as seen in this case, should be considered as an etiology of aortitis in cases with suggestive history and radiological findings.
